# Factors associated with adolescent use of tobacco products in the Upper East Region of Ghana: A cross-sectional study

**DOI:** 10.18332/tid/215181

**Published:** 2026-01-29

**Authors:** Divine Darlington Logo, Prakash B. Kodali, Judith Anaman-Torgbor, Benjamin W. Chaffee, Pamela M. Ling, Stella Bialous, Ellis Owusu-Dabo

**Affiliations:** 1 Research and Development Division, Ghana Health Service, Accra, Ghana; 2 Center for Tobacco Control Research and Education, University of California, San Francisco, United States; 3 Department of Public Health and Community Medicine, Central University of Kerala, Kerala, India; 4 Department of Public Health Nursing, School of Nursing and Midwifery, University of Health and Allied Sciences, Ho, Ghana; 5 School of Dentistry, University of California, San Francisco, United States; 6 Division of General Internal Medicine, Department of Medicine, University of California, San Francisco, United States; 7 School of Public Health, College of Health Sciences, Kwame Nkrumah University of Science and Technology, Kumasi, Ghana

**Keywords:** prevalence, single-tobacco use, multiple tobacco use, adolescents, Upper East Region of Ghana

## Abstract

**INTRODUCTION:**

Tobacco use among adolescents is a concern in the Upper East Region of Ghana. We estimated the prevalence and identified factors contributing to single and multiple use of tobacco products among junior high school students in Ghana.

**METHODS:**

We conducted a cross-sectional analysis of a baseline survey of a school-based tobacco control intervention among adolescents in the Upper East Region of Ghana in 2022. A multi-stage cluster sampling approach was employed to identify the study sample, and data were collected using self-administered questionnaires. Current use of single tobacco products (at least one: cigarette, e-cigarette, shisha, or smokeless tobacco products) and multiple products (≥2 products) in the past 30 days was assessed. Multinomial logistic regression was used to assess the association of sociodemographic characteristics, perceptions towards tobacco’s health risks, and exposure to tobacco products with single and multiple product use. Adjusted relative risk ratios (ARRR) and their corresponding 95% confidence intervals (CI) were computed.

**RESULTS:**

We surveyed 1328 adolescents, comprising an equal proportion of males (49.8%) and females (50.4%). One in five (21.7%) reported using tobacco products, with 11.5% using single products and 13.0% using multiple products. Shisha (13.6%), cigarettes (10.6%), e-cigarettes (8.2%), and smokeless tobacco (6.0%) were used. A number of factors were identified to be associated with tobacco use among adolescents.

**CONCLUSIONS:**

One in five junior high school students used at least one form of tobacco product. Adolescent tobacco use is impacted by demographic factors and risk perceptions. Further studies are needed to better understand these associations.

## INTRODUCTION

Tobacco use remains a significant public health challenge worldwide, killing more than 8 million people each year, with 170.9 million disability-adjusted life-years lost attributed to tobacco use in 2017^1^. Adolescents are at a heightened risk of initiating tobacco consumption during their formative years^2^, making them the prime target for the tobacco industry’s marketing tactics^3^. A review of Global Youth Tobacco Surveys (GYTS) in 131 countries (1999–2005) found that 8.9% of adolescents aged 13–15 years smoked cigarettes, and 11.2% used other tobacco products in the past 30 days^4^. Global School-based Student Health Surveys reported similar results for 68 low- and middle-income countries (LMICs) (2006–2013)^5^.

The overall prevalence of cigarette smoking among adolescents in Ghana is low at 2.8%^6,7^ relative to other regions in Sub-Saharan Africa^8^ which ranged from 5.4% cigarette use prevalence in Tanzania to 36.4% in Seychelles^9^. The northern zone of Ghana, which includes the Upper East Region, reports 28.3% of tobacco use prevalence among adolescents compared to 8.9% nationally^6,10^. Understanding the factors that drive tobacco use in this region is crucial because adolescence is a high-risk period in which behaviors learned may carry over into adulthood, with associated long-term health consequences^11^. Previous studies have highlighted a growing trend in the use of various tobacco products among adolescents in the northern zones of Ghana including the Upper East Region, beyond conventional cigarettes (7.6%), including smokeless tobacco (9.1%), waterpipe (shisha) (7.8%), and electronic cigarettes (22.6%), raising concerns about nicotine dependence and adverse health outcomes^10,12^.

Data from the Global Youth Tobacco Survey (GYTS) revealed that multiple sociodemographic and environmental factors influence tobacco use among Ghanaian adolescents^12^. Of specific interest, predictors such as age, gender, possession of pocket money, secondhand smoke within households, and knowledge about tobacco have been closely associated with the likelihood of tobacco consumption^6,10^. The Upper East Region, where socioeconomic underdevelopment is common and cultural environments are unique, has patterns of tobacco consumption^13^ that should be carefully investigated. For example, exposure to tobacco advertising, peer and family influences, and regional disparities contribute to the complex landscape of adolescent tobacco use in this setting^7^.

Tobacco use is culturally rooted among the people of the Upper East Region of Ghana. This is because tobacco is significant in marriage and funeral ceremonies, freely cultivated as a cash crop, and used in almost every home in the region^13,14^. Despite the existence of policies to control commercial tobacco use, like bans on advertisements and laws against smoking in public places, and sales to minors^15,16^, their enforcement in the Upper East Region is inadequate, and focused interventions are lacking^6^. Identification and understanding of the factors unique to the use of tobacco among adolescents in the Upper East Region is needed to design effective prevention and cessation interventions.

Previous studies on adolescent tobacco use in Ghana have drawn on nationally representative data, including the GYTS and data from the Ministry of Gender, Children, and Social Protection, Ghana^6,10,17^. While these studies provided valuable information, they are limited by their broad scope, which may mask regional inequalities.

This study aimed to address these gaps by collecting primary data and determining prevalence, associated factors of single and multiple tobacco product use, environmental exposure, and risk perceptions among adolescents in the Upper East Region of Ghana. This study further provides region-specific data on adolescent tobacco use, which may support the development of tailored interventions to improve public health outcomes by preventing and reducing tobacco-related deaths and diseases.

## METHODS

### Study population

This study presents baseline cross-sectional findings from the Smart-Kids trial, conducted in 2022, in the Upper East Region of Ghana. Smart-Kids used a randomized controlled trial design to develop and test a school-based intervention aimed at enhancing refusal skills, improving self-esteem, and increasing knowledge about the risks associated with tobacco use, potentially reducing smoking initiation and increasing the quit rate among adolescents. The study was conducted among 12 schools in Builsa South and Talensi districts in the Upper East Region of Ghana. The two districts share similar demographic characteristics, such as a high proportion of young population and a rural setting, with the majority of the population engaged in agriculture as their primary source of livelihood^18^. Public and private schools that were officially registered with the Ghana Education Service were invited to participate. Schools were eligible if they had an enrollment of 60 or more, had not implemented any tobacco control programs within 12 months preceding the survey, or did not receive support from the tobacco industry. All students from the selected schools who were within the adolescent age group (10–19 years) met the inclusion criteria for the randomized trial; however, all students from the selected schools were eligible to participate in the survey. We received ethical clearance from the Committee on Human Research, Publications, and Ethics (CHRPE) at Kwame Nkrumah University of Science and Technology (Approval number: CHRPE/AP/179/21; Date 10-05-2021). We obtained informed consent from parents and verbal assent from students, emphasizing that participation was voluntary, anonymous, and that participants could withdraw at any time or decline to answer specific questions.

### Sample size estimation

The estimated sample size for this analysis was based on the trial, and the required sample size needed to observe an interventional impact of 4.6% was 1302 participants. For the present observational analysis of baseline trial data, assuming a 5.0% significance level (alpha), an anticipated overall prevalence of tobacco use of 7.6%^6^, and a clustering-related design effect of 1.59, with a precision of ±2% (95% confidence interval width) and 80.0% power to detect a relative risk as small as 2.1 when one-third of the sample is in an ‘exposed’ group and the comparison prevalence of tobacco use is 5.0%.

### Sampling procedure

A multi-stage sampling approach was employed to select the study participants. Firstly, the Upper East Region was selected due to its high tobacco use among adolescents^6^. Next, two districts were randomly chosen from the 15 in the region. Within these districts, 42 and 24 junior high schools were identified, respectively. Schools with enrollment sizes of ≥60 students were selected using probability proportional to enrollment size. Twelve schools (six from each district) were randomly selected, yielding a total number of 1328 students. Finally, all students in the selected schools were eligible to participate in the study.

### Study measures

We employed a self-administered survey questionnaire developed by adapting the items from the GYTS survey questionnaire^19^ and review of literature on tobacco use among youth^20^. Content and face validity of the questionnaire were ensured through consultations with the experts within and outside the study team. The questionnaire included sections that captured 1) participants’ past 30-day tobacco use; 2) perceptions of harm caused by tobacco; and 3) exposure to smoking-related behaviors, and 4) sociodemographic covariates. To assess the tobacco use, the participants were asked: ‘During the past 30 days, how often did you use any of these tobacco products (cigarettes, shisha, e-cigarettes, and smokeless tobacco)?’. These questions were asked separately for each tobacco product, with responses: 1) once a week; 2) at least once a week but not every day; 3) every day; 4) I did not use the tobacco product during the past 30 days; and 5) I have never tried tobacco products. Current use was defined as use of any tobacco product (cigarette, shisha, e-cigarette, smokeless tobacco) at least once a week (including once a week, at least once a week but not every day, and every day); whereas those reporting not using the product(s) in the past 30 days, and never trying the products were coded together as non-use.

The items capturing individual tobacco product use were pooled to develop a single outcome variable ‘current tobacco use’ with three responses 1) current use of single tobacco products (at least one of cigarette, e-cigarette, shisha, or smokeless tobacco products) in the past 30 days, and 2) current use of multiple products (two or more products) in the past 30 days; and 3) no use.

Participants’ perceptions of tobacco harm were assessed based on four items: 1) ‘In case you decide to smoke, do you think you will become addicted?’; 2) ‘In case you decide to smoke, do you think you will become sick with tobacco-related diseases like heart disease, stroke, and cancer?’, 3) ‘Do you know that continuous use of tobacco causes lung cancer’; and 4) ‘Do you know tobacco causes heart disease’. Each of these items were measured on a 4-point Likert scale, ranging from: 1=strongly disagree to 4=strongly agree, which were coded into binary levels as ‘disagree’ (strongly disagree and disagree) or as ‘agree’ (agree and strongly agree).

The participants’ exposure to smoking-related behaviors was assessed based on three binary items: 1) having a household member who uses tobacco; 2) ever being sent on a tobacco errand like buying or selling cigarettes; and 3) being asked to light a cigarette or any other tobacco product by others.

The sociodemographic covariates were self-reported by the individual and included: 1) gender (male, female); 2) age (as categorical variable); 3) junior high school grade (Grade 1, Grade 2, Grade 3, corresponding to Forms 1–3); 4) religion (Christian, Muslim, Traditionalist, No religion); 5) Ethnicity (Bulsa, Talensi, Frafra and other); and 6) parents occupation (self-employed, formal employment, and unemployed).

### Statistical analysis

We employed descriptive statistics (i.e. frequencies and percentages) to characterize the study sample. Prevalence estimates were calculated for the use of each tobacco product (cigarette, shisha, smokeless tobacco, and e-cigarette), single-use and multiple-use of tobacco products. We also estimated the proportions of those who use single products versus those who use multiple products, across key demographic subgroups and study variables.

We constructed bivariate tables to examine the associations of the variable ‘current tobacco use’ with the responses single, multiple, and no-use with: 1) participants’ perception towards harms of smoking, 2) participants’ exposure to smoking-related behaviors, and 3) sociodemographic covariates. We further expanded this analysis with a multivariable analysis, in which we developed a multinomial logistic regression model comparing single tobacco use and multiple tobacco product use to the reference category of ‘no tobacco use’. The independent variables in the model included: 1) gender, 2) junior high school grade, 3) religion, 4) ethnicity, 5) parents’ occupation, 6) agree tobacco makes you addicted, 7) agree tobacco makes you sick, 8) agree tobacco causes heart disease, 9) agree tobacco causes lung cancer, 10) household member uses tobacco, 11) ever sent on a tobacco errand, and 12) ever asked to light a cigarette or any tobacco product. Age of the participant showed strong multicollinearity with the ‘junior high school grade’ and was excluded from the model. Considering the sampling involved clusters (i.e. schools from which the participants were sampled), we adjusted for cluster differentials using robust standard errors. We computed the adjusted relative risk ratios (ARRR) and their corresponding 95% confidence intervals (CIs) to report associations. Two different sets of coefficients, single tobacco use and multiple tobacco use, were reported as the analysis was a multinomial logistic regression

The data cleaning and analysis were conducted using Stata Version 18 software (StataCorp LLC, Texas, USA). The analysis was two-sided, and the significance level was set at p<0.05 to determine statistical significance.

### Ethics and administrative approval

Administrative approval was obtained from the Upper East Regional Education Service Directorate and the two districts for this study. The Committee on Human Research, Publications, and Ethics (CHRPE) at the Kwame Nkrumah University of Science and Technology reviewed and approved the study protocol (Protocol approval number: CHRPE/AP/179/21; Date: 10.05.2021). Parental consent and assent from students were sought before data collection commenced. Students were also informed that participation was voluntary and, they could discontinue when they wished or decide not to respond to a question that they were not comfortable with.

## RESULTS

The study sample comprised 1328 participants from 12 schools, with 50.2% being females. The mean age was 15.2 years (SD=1.6) and about 40.0% were in junior high school grade 1. More than two-thirds (73.3%) were Christians, and Bulsa (46.9%) and Talensi (29.2%) were the dominant ethnic groups. More than half (50.2%) of the participants reported that their parents were self-employed (Table 1).

**Table 1:** Sociodemographic characteristics of participants in junior high schools in the Upper East Region of Ghana, 2022 (N=1328)

*Characteristics*	*Overall^#^* *n (%)*	*Non-use of tobacco* products* %	*Single product* use* %	*Multiple product* use* %
**Gender**				
Male	662 (49.8)	75.5	11.5	13.0
Female	666 (50.2)	81.1	9.9	9.0
**Age** (years)				
10––12	42 (3.2)	85.7	4.8	9.5
13–15	787 (59.3)	78.9	10.4	10.7
16–18	465 (35.0)	76.6	11.6	11.8
≥19	34 (2.6)	79.4	11.8	8.8
**Junior high school**				
Grade 1	498 (37.5)	77.5	9.4	13.1
Grade 2	441 (33.2)	77.6	12.2	10.2
Grade 3	389 (29.3)	80.2	10.5	9.3
**Religion**				
Christian	974 (73.3)	81.4	9.2	9.3
Muslim	102 (7.7)	77.5	13.7	8.8
Traditionalist	137 (10.3)	62.8	13.1	24.1
No religion	115 (8.7)	72.4	16.3	11.3
**Ethnicity**				
Frafra and other	317 (23.9)	70.7	13.6	15.8
Talensi	388 (29.2)	82.0	8.8	9.3
Bulsa	623 (46.9)	79.9	10.4	9.6
**Parent’s occupation**				
Self-employed	666 (50.2)	79.7	9.6	10.7
Formal employment	246 (18.5)	70.3	15.4	14.2
Unemployed	416 (31.3)	80.8	9.6	9.6
**Tobacco makes you addicted**				
Disagree/did not respond	766 (57.7)	78.6	10.4	11.0
Agree	562 (42.3)	77.9	11.0	11.0
**Tobacco makes you sick**				
Disagree/did not respond	599 (45.1)	72.0	13.5	14.5
Agree	729 (54.9)	83.5	8.4	8.1
**Tobacco causes heart disease**				
Disagree/did not respond	464 (34.9)	71.8	13.6	14.7
Agree	864 (65.1)	81.8	9.1	9.0
**Tobacco causes lung cancer**				
Disagree/did not respond	518 (39.0)	73.0	12.2	14.9
Agree	810 (61.0)	81.7	9.8	8.5
**Household member use of tobacco**				
No	1095 (82.5)	78.2	10.9	11.0
Yes	233 (17.5)	79.0	9.9	11.2
**Ever sent on tobacco errand**				
No	1039 (78.2)	78.5	11.3	10.2
Yes	289 (21.8)	77.5	8.7	13.8
**Ever sent to light cigarette or any tobacco product**				
No	1133 (85.3)	78.7	10.9	10.4
Yes	195 (14.7)	75.9	9.7	14.4

# Overall column percentages are presented for the total sample (N=1328).

* Row percentages are presented for non-use, single-product use, and multiple-use groups.

### Tobacco use among participants

**Figure 1:**
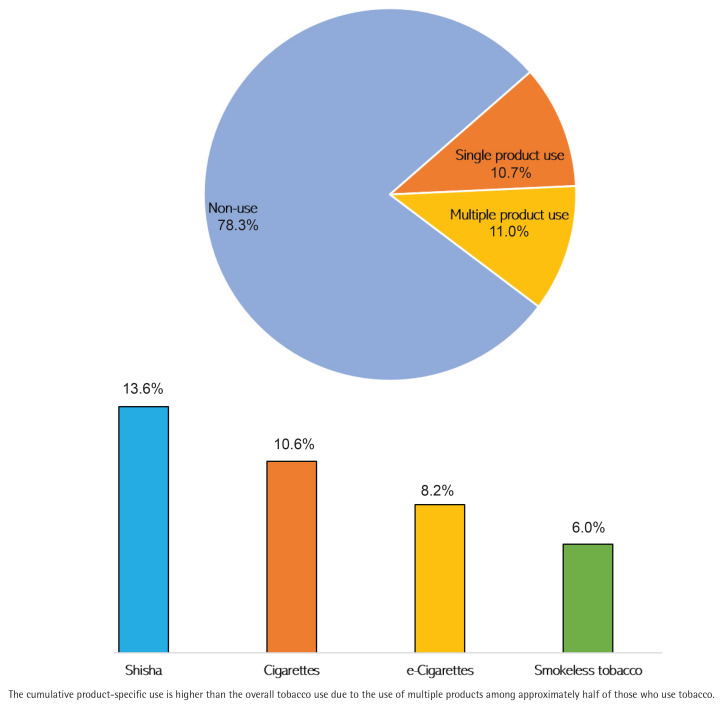
Prevalence of single, multiple, and product-specific tobacco use among the adolescents in Upper East Region of Ghana (N=1328)

A majority of the study participants (78.3%) do not use tobacco products, while 21.7% reported current use of any tobacco products. Additionally, 10.7% (95% CI: 9.1–12.4) used single products, and 11.0% (95% CI: 9.4–12.8) used multiple products. Shisha (13.6%; 95% CI: 11.9–15.5) was the most commonly used tobacco product, followed by cigarettes (10.6%; 95% CI: 9.0–12.3), e-cigarettes (8.2%; 95% CI: 6.8–9.8), and smokeless tobacco (6.0%; 95% CI: 4.8–7.4) (Figure 1).

Participants in Grade 2 (12.2%) had a slightly higher proportion of single tobacco use compared to Grade 1 (9.4%). However, considering multiple tobacco product use, those in Grade 1 (13.1%) were proportionally higher than Grade 2 (10.2%) and Grade 3 (9.3%). Participants reporting their parents to be in formal employment had a higher proportion of single tobacco product use (15.4%) and multiple tobacco product use (14.2%).

A greater proportion of males (13.0%) compared to females (9.0%) used multiple tobacco products. Similarly, more participants who identified as Traditionalists (24.1%) compared to Christians (9.3%) used multiple products. Ethnic differences were observed, with individuals belonging to the Frafra group reporting a higher proportion of single (13.6%) and multiple use (15.8%) compared to others.

More participants who agreed that tobacco makes one sick were non-users (83.5%) while among those who disagreed 13.5% used a single product, and 14.5% used multiple products; similar observations were made with regard to those disagreeing with the statements ‘tobacco causes heart disease’ [single use (13.6%), multiple use (14.7%)] and ‘tobacco causes lung cancer’ [single use (12.2%), multiple use (14.9%)] (Table 1). A higher proportion of multiple product use was observed among the participants who reported being sent on tobacco errands (13.8%), and those who were ever asked to light a cigarette or any tobacco product (14.4%).

### Factors associated with tobacco use among the participants

Table 2 presents a multinomial regression analysis of factors associated with tobacco use among adolescents. The relative risk ratio for multiple tobacco use was nearly three times higher among participants who practiced Traditional religion (ARRR=2.89; 95% CI: 1.82–4.60) compared to Christians. Adolescents whose parents were in formal employment were more likely to use single tobacco products (ARRR=1.77; 95% CI: 1.08–2.89) compared to those whose parents were self-employed. Participants who agreed that ‘tobacco makes you sick’ were less likely to use single (ARRR=0.52; 95% CI: 0.34–0.80), and multiple tobacco products (ARRR=0.50; 95% CI: 0.31–0.82) compared to those who disagreed (Table 2).

**Table 2:** Multinomial regression of factors associated with tobacco use among adolescents in junior high schools, Upper East Region, Ghana, 2022 (N=1328)

*Variables*	*Single tobacco use*		*Multiple tobacco use*	
	*ARRR (95% CI)*	*p*	*ARRR (95% CI)*	*p*
**Gender**				
Male (ref.)				
Female	0.88 (0.67–1.6)	0.375	0.83 (0.49–1.41)	0.501
**Junior high school**				
Grade 1 (ref.)				
Grade 2	1.18 (0.51–2.71)	0.442	0.72 (0.18–3.00)	0.656
Grade 3	1.05 (0.51–2.16)	0.889	0.72 (0.22–2.36)	0.590
**Religion**				
Christian (ref.) Muslim	1.52 (0.99–2.33)	0.058	1.00 (0.51–1.97)	0.994
Traditionalist	1.58 (0.92–2.72)	0.097	2.89 (1.82–4.60)	<0.001
No religion	1.99 (0.96–4.14)	**0.065**	1.30 (0.54–3.14)	0.561
**Ethnicity**				
Frafra and other (ref.)				
Talensi	0.64 (0.17–2.39)	0.505	0.60 (0.11–3.37)	0.563
Bulsa	0.63 (0.19–2.09)	0.453	0.62 (0.13–2.90)	0.544
**Parent’s occupation**				
Self-employed (ref.)				
Formal employment	1.77 (1.08–2.89)	**0.023**	1.61 (0.82–3.14)	0.164
Unemployed	1.04 (0.70–1.56)	0.876	1.12 (0.68–1.83)	0.661
**Tobacco makes you addicted**				
Disagree/did not respond (ref.)				
Agree	1.57 (0.88–2.79)	0.126	1.68 (0.96–2.95)	0.069
**Tobacco makes you sick**				
Disagree/did not respond (ref.)				
Agree	0.52 (0.34–0.80)	**0.003**	0.50 (0.31–0.82)	**0.006**
**Tobacco causes heart disease**				
Disagree/did not respond (ref.)				
Agree	0.69 (0.36–1.32)	0.261	0.76 (0.46–1.25)	0.279
**Tobacco causes lung cancer**				
Disagree/did not respond (ref.)				
Agree	1.05 (0.71–1.56)	0.807	0.69 (0.41–1.18)	0.180
**Household member use of tobacco**				
No (ref.)				
Yes	1.05 (0.56–1.99)	0.8876	0.99 (0.58–1.68)	0.965
**Ever sent on tobacco errand**				
No (ref.)				
Yes	0.73 (0.40–1.34)	0.312	1.26 (0.81–1.99)	0.308
**Ever asked to light cigarette or any tobacco product**				
No (ref.)				
Yes	1.07 (0.65–1.76)	0.7887	1.19 (0.74–1.92)	0.473

Dependent variable: Tobacco use: do not use (reference), single tobacco product use, multiple tobacco products use. ARRR: adjusted relative risk ratio.

Also, there is suggestive evidence that being a Muslim (ARRR=1.52; 95% CI: 0.99–2.33) and having no religion (ARRR=1.99; 95% CI: 0.96–4.14) may be associated with higher odds of single tobacco use compared to Christians. Also, adolescents who are from the Traditional religion (ARRR=1.58; 95% CI: 0.92–2.72) may be associated with an increased likelihood of single tobacco use compared to Christians. However, these associations were not statistically significant (Table 2).

## DISCUSSION

This study examined factors associated with single and multiple use of tobacco among adolescents in junior high schools in the Upper East Region of Ghana. Religious affiliation, parental occupation, and knowledge about tobacco’s health risks were significantly associated with single and multiple use of tobacco products. However, other variables, such as gender, grade level, and household member tobacco use, did not show any significant associations with tobacco use in this population. The findings suggest that the relationship between religious inclination and tobacco use, as well as the impact of knowledge about tobacco harms, needs to be investigated. Further studies, such as cohort or intervention studies, could explore the benefits of engaging religious organizations in tobacco control efforts and the effectiveness of health promotion materials aimed at adolescents in the Upper East Region and other vulnerable populations. This finding is consistent with studies that suggest religiosity serves as a protective factor against tobacco use, with certain religious affiliations associated with lower odds of tobacco use^21,22^. Studies examining the association between tobacco use and religion are scarce in the Sub-Saharan region. However, a study conducted among university students in South Africa found that higher religiosity was associated with lower tobacco use^23^. Findings from this study align with another Ghanaian study that linked low tobacco use to health policies, anti-smoking campaigns, cultural and religious factors^24^ emphasizing the potential role of religious intervention prevention programs in protecting public health.

We found that adolescents with parents in formal employment were more likely to engage in single tobacco use. Our findings disagreed with studies suggesting that parental unemployment negatively affects adolescent life by reducing parental support and health-protective effects, potentially predisposing the child to deviant behavior, including smoking uptake^25,26^. A Previous study found that children of parents working in manual labor or service industry jobs (blue-collar workers) have a higher likelihood of smoking compared to children of parents to working in office or professional settings (white-collar workers)^26^, which is in contrast to our findings. Another finding suggests that parents with non-formal work, particularly in low-income families, may experience more pronounced consequences, potentially predisposing adolescents to deviant behavior, including smoking initiation^27^. These disagreements may be attributed to several factors. The possibility of formal workers in our setting having long working hours and contributing to less parental supervision at home among their wards, may lead to tobacco use^28^. Formal employment might also influence socioeconomic factors such as increased disposable income, potentially increasing their ward’s access to and likelihood of purchasing tobacco products^29^. Cultural and environmental factors specific to our study population may also play a role. Further research is needed to explore these potential explanations and understand the complex relationships between parental occupation and adolescent tobacco use.

Adolescents who believed that tobacco use leads to illness were less likely to use single or multiple tobacco products, suggesting that awareness of the harmful effects of tobacco may deter use. Adolescents who recognize the addictive nature of tobacco and yet decide to use it may rationalize their behavior by focusing on perceived barriers to quitting over the benefits when they quit^30^.

In line with another study^31^, adolescents who use tobacco and intend to quit tend to underestimate their risk of smoking-related harm and addiction, while those who acknowledge tobacco’s addictive nature are likely to smoke, potentially due to this optimism bias. There is also evidence that adolescents understand cigarette addiction but lack clarity on what addiction involves, particularly the challenges of quitting and the tendency for sustained smoking despite their desires^32^. Our study has found that awareness and attitudes are associated with adolescent tobacco use. More than two-thirds of participants who do not use any form of tobacco product acknowledged that tobacco causes sickness and linked tobacco to serious health risks, including lung cancer and heart disease. These findings indicate that community education campaigns, in conjunction with other influences, have probably enhanced awareness of the dangers of tobacco, which may have an impact on patterns of tobacco use.

Tobacco use among adolescents is multifaceted, with religion, parental occupation, and attitudes to tobacco risks all playing a part. Our research identifies the necessity for tailored interventions to deter the uptake of smoking and promote healthy lifestyles for young people. There is evidence that adolescents who are better informed about the health risks of tobacco are less inclined to smoke and more inclined to quit^33^. Therefore, health-focused anti-tobacco education and effective communication programs need to be included in regular school health curricula and other adolescent interventions. Further research is needed to better understand more about particular attitudes and beliefs that lead adolescents to smoke. This will aid policymakers and healthcare practitioners in devising effective, focused prevention and intervention programs that will promote healthier choices.

### Limitations

We acknowledge some limitations of our study, including the possibility of social desirability bias, cross-sectional design, and recall bias. The analysis relies on self-reported data, which may be liable to social desirability bias, where participants may provide biased responses due to societal pressures or stigma surrounding tobacco use. The cross-sectional design means that it provides a snapshot of adolescent tobacco use at a single point in time, limiting the ability to establish causality between variables. The recall bias may influence the accuracy of self-reported data, as participants’ recollections of past experiences or behaviors may be inaccurate. We mitigated these issues by maintaining the anonymity of responses, using validated questionnaires, and posing direct questions. However, residual confounding due to unmeasured or controlled factors, such as socioeconomic status, exposure to tobacco advertisements, and peer influence, may be present, and our results should be interpreted with these limitations in mind. Given that our study was conducted in only one region of the country, we acknowledge that the results may not be generalizable to other settings and that future studies should consider replicating our study in other settings to enhance its generalizability.

## CONCLUSIONS

Our research determines the multifaceted causes of tobacco smoking among adolescents, such as religion, parents’ occupation, and attitudes toward danger from tobacco. The findings assert the need for further studies, particularly longitudinal studies, to better understand the dynamics of tobacco use among adolescents. These additional studies will provide evidence for effective policies and interventions to prevent smoking uptake and promote healthier lifestyles among adolescents. Policymakers and health professionals can utilize these data to address the unique needs of different groups of adolescents and reduce the burden of tobacco use.

## Data Availability

The data supporting this research are available from the authors on reasonable request.
